# Bloqueio Atrioventricular de 1º Grau: Um Achado nem Sempre Benigno!

**DOI:** 10.36660/abc.20220643

**Published:** 2022-10-05

**Authors:** Tan Chen Wu

**Affiliations:** 1 Hospital das Clínicas Faculdade de Medicina Universidade de São Paulo São Paulo SP Brasil Instituto do Coração (InCor) – Unidade de Arritmia - Hospital das Clínicas da Faculdade de Medicina da Universidade de São Paulo , São Paulo , SP – Brasil

**Keywords:** Bloqueio Atrioventricular, Mortalidade, Insuficiência Cardíaca

O bloqueio atrioventricular (BAV) de 1º grau se caracteriza pela presença de ritmo sinusal, condução AV 1:1 e intervalo PR > 200ms. A prevalência varia de acordo com a faixa etária, sendo relativamente rara na população < 60 anos (1%), com aumento para 6% nos indivíduos > 60 anos. A prevalência descrita na população em geral varia de 2 a 14%. ^1^ Na maioria dos casos (75%), é devido a um bloqueio proximal ou nodal que tende a melhorar a condução com redução do intervalo PR com manobras que levam ao aumento do tônus adrenérgico e/ou infusão de atropina. ^2^

Habitualmente considerado como o achado benigno, o prolongamento do intervalo PR ou BAV de 1º grau tem seu prognóstico questionado mais recentemente, por evidências de ser o fator independente no aumento do risco de fibrilação atrial (FA), de implante de marca-passo cardíaco ^3^ e de mortalidade de todas as causas. Na coorte de Framingham, ^4^ a presença de BAV 1º grau é considerada um fator de risco para o desenvolvimento de FA, fato confirmado em estudos subsequentes em outras bases populacionais com a demonstração da associação entre prolongamento do PR e a insuficiência cardíaca e/ou FA. ^5^

A relação do BAV de 1º grau com desfecho desfavorável também foi observada em pacientes com cardiopatia estrutural em uma coorte descrita por Higuchi et al. em 414 pacientes com cardiomiopatia hipertrófica (CMH). Aproximadamente 1/4 da coorte demonstrou prolongamento do intervalo PR ≥200ms, que se associou em análises multivariadas com morte relacionada à CMH (RR ajustado 2,41; 95% CI, 1.27–4.58), e desfecho combinado de morte súbita ou eventos arrítmicos potencialmente letais (RR ajustado 2,60; 95% CI, 1,28–5,2). ^6^

O fato vem acrescido do reconhecimento nos últimos anos de cardiomiopatia atrial, com implicação prognóstica, principalmente em pacientes portadores de FA. Um dos fatores etiológicos, a inflamação, base para diversos processos patológicos, tem o seu papel cada vez mais definido no remodelamento atrial, o qual pode ser consequência ou reflexo das doenças sistêmicas e metabólicas como hipertensão arterial sistêmica, diabetes mellitus, insuficiência renal, apneia do sono e obesidade além dos processos locais como estiramento atrial, infarto do miocárdio e fatores genéticos. ^7 , 8^ A reação inflamatória que envolve estresse oxidativo, alterações na regulação do cálcio, produção de citocinas pró-inflamatórias, proliferação de fibroblastos, miofibroblastos e matriz extracelular e a apoptose causa a fibrose atrial, revelada no eletrocardiograma pelo prolongamento do intervalo PR ou BAV e aumento do diâmetro e volume atrial no ecocardiograma. ^9^

Com o objetivo de avaliar o fator prognóstico de todos os BAV em uma população latina, Paixão et al. do estudo CODE ( *Clinical Outcomes in Digital Electrocardiology* ), avaliaram a associação entre o BAV e mortalidade geral em uma coorte brasileira de atenção primária, com 1.557.901 pacientes, em um seguimento médio de 3,7 anos, baseado em banco de dados com eletrocardiogramas realizados majoritariamente em unidades básicas de saúde. Destes, 40% eram homens e a idade média foi de 51 anos. A prevalência de BAV foi de 1,38%, a maioria de 1º grau (1,32% - 20.644), com 0,02% (273) e 0,04% (621) de 2º e 3º grau respectivamente. Os pacientes com BAV de 1º, 2º e 3º graus foram associados a uma taxa de sobrevida de 24% (RS= 0,76; IC de 95%: 0,71 a 0,81; p < 0,001), 55% (RS = 0,45; IC de 95%: 0,27 a 0,77; p = 0,01) e 64% (RS = 0,36; IC de 95%: 0,26 a 0,49; p < 0,001) menor quando comparados ao grupo controle, respectivamente, e que somente os pacientes com BAV 2º grau Mobitz I (212 pacientes), na análise de sobrevida dividida por subtipo de BAV, não foram associados a maior mortalidade, ao contrário dos pacientes com BAV 2:1 (61 pacientes), com taxa de sobrevida de 79% menor do que o grupo controle. Além de confirmar o pior prognóstico, com a menor sobrevida, nos pacientes com BAV 2º grau (exceto os de Mobitz I) e 3º grau, o estudo reafirmou a redução de sobrevida também em pacientes com BAV de 1º grau. ^10^ Vale ressaltar que a média de idade foi semelhante a outros estudos (56 anos) que mostraram desfechos semelhantes em relação ao BAV de 1º grau em revisão sistemática e metanálise realizado por Kwok et al. ^1^ com 400.750 paciente na qual observou o aumento do risco relativo de 1,24 (95% CI 1.02-1.51) para mortalidade, 1,39 (95% CI 1.18-1.65) para insuficiência cardíaca e 1,45 (95% CI 1.23-1.71) para FA. Curiosamente, não houve aumento de mortalidade cardiovascular nesta metanálise, dado não avaliado pelo estudo CODE. Outra particularidade na coorte brasileira é a presença relativamente frequente de doença de Chagas, causa frequente de BAV.

Com as evidências atuais, o BAV de 1º grau deve ser visto com mais atenção e o eletrocardiograma, apesar de todo o avanço na cardiologia, com exames diagnósticos por imagens cada vez mais detalhados e específicos, permanece como uma ferramenta simples, disponível, útil e fundamental na nossa rotina.

## References

[1] Kwok CS, Rashid M, Beynon R, Barker D, Patwala A, Morley-Davies A (2016). Prolonged PR Interval, First-degree Heart Block and Adverse Cardiovascular Outcomes: A Systematic Review and Meta-analysis. Heart.

[2] Kusumoto FM, Schoenfeld MH, Barrett C, Edgerton JR, Ellenbogen KA, Gold MR (2019). 2018 ACC/AHA/HRS Guideline on the Evaluation and Management of Patients with Bradycardia and Cardiac Conduction Delay: A Report of the American College of Cardiology/American Heart Association Task Force on Clinical Practice Guidelines and the Heart Rhythm Society. Heart Rhythm.

[3] Lewalter T, Pürerfellner H, Ungar A, Rieger G, Mangoni L, Duru F (2018). “First-degree AV block-a benign entity?” Insertable Cardiac Monitor in Patients with 1st-degree AV Block Reveals Presence or Progression to Higher Grade Block or Bradycardia Requiring Pacemaker Implant. J Interv Card Electrophysiol.

[4] Schnabel RB, Sullivan LM, Levy D, Pencina MJ, Massaro JM, D’Agostino RB (2009). Development of a Risk Score for Atrial Fibrillation (Framingham Heart Study): A Community-based Cohort Study. Lancet.

[5] Magnani JW, Wang N, Nelson KP, Connelly S, Deo R, Rodondi N (2013). Electrocardiographic PR Interval and Adverse Outcomes in Older Adults: The Health, Aging, and Body Composition study. Circ Arrhythm Electrophysiol.

[6] Higuchi S, Minami Y, Shoda M, Shirotani S, Saito C, Haruki S (2020). Prognostic Implication of First-Degree Atrioventricular Block in Patients With Hypertrophic Cardiomyopathy. J Am Heart Assoc.

[7] Harada M, Nattel S (2021). Implications of Inflammation and Fibrosis in Atrial Fibrillation Pathophysiology. Card Electrophysiol Clin.

[8] Vyas V, Hunter RJ, Longhi MP, Finlay MC (2020). Inflammation and Adiposity: New Frontiers in Atrial Fibrillation. Europace.

[9] Szilágyi J, Sághy L (2021). Atrial Remodeling in Atrial Fibrillation. Comorbidities and Markers of Disease Progression Predict Catheter Ablation Outcome. Curr Cardiol Rev.

[10] Paixão GMM, Lima EM, Quadros AB, Cabral DPR, Coelho RR, Oliveira DM (2022). Association between Atrioventricular Block and Mortality in Primary Care Patients: The CODE Study. Arq Bras Cardiol.

