# Supplementary material for: Bloqueio Atrioventricular de 1º Grau: Um Achado nem Sempre Benigno!
Source: Arq Bras Cardiol. 2022 Oct 5;119(4):572–3. [Article in Portuguese] doi: 10.36660/abc.20220643 (PMC9563881; doi:10.36660/abc.20220643)
Supplement: Translation

# Translation: First-Degree Atrioventricular Block: A Finding Not Always Benign!

**Authors:** Tan Chen Wu

**Affiliations:** 1 Brazil Instituto do Coração (InCor) – Unidade de Arritmia - Hospital das Clínicas da Faculdade de Medicina da Universidade de São Paulo, São Paulo, SP – Brazil

**Keywords:** Atrioventricular Block, Mortality, Heart Failure

First-degree atrioventricular block (AVB) is characterized by sinus rhythm, AV conduction 1:1 and PR interval > 200ms. The prevalence varies according to age group, relatively rare in the population < 60 years (1%), with an increase to 6% in individuals > 60 years. The reported prevalence in the general population ranges from 2 to 14%.^1^ In most cases (75%), it is due to a proximal or nodal block that tends to improve conduction with a reduction in the PR interval with maneuvers that lead to an increase in adrenergic tone and/or atropine infusion.^2^

Usually considered a benign finding, PR interval prolongation or first-degree AV block has its prognosis more recently questioned due to emerging evidence that it is the independent factor in the increased risk of atrial fibrillation (AF), cardiac pacemaker implantation^3^ and all-cause mortality. In the Framingham cohort,^4^ the presence of first-degree AV block is considered a risk factor for the development of AF, a fact confirmed in subsequent studies in other community-based cohort with the demonstration of the association between PR prolongation and heart failure and/or AF.^5^

The relationship between first-degree AVB and the unfavorable outcome was also observed in patients with structural heart disease in a cohort described by Higuchi et al. in 414 patients with hypertrophic cardiomyopathy (HCM). Approximately 1/4 of the cohort demonstrated PR interval prolongation ≥200ms, which was associated in multivariate analyzes with HCM-related death (adjusted RR 2.41;95%CI, 1.27–4.58), and the potentially lethal arrythmic endpoint of sudden death or life-threatening arrhythmic events (adjusted RR 2.60;95% CI, 1.28–5.2).^6^

This fact is compounded by the recognition in recent years of atrial cardiomyopathy, with prognostic implications, especially in patients with AF. One of the etiological factors, inflammation, the basis for several pathological processes, has its role increasingly defined in atrial remodeling, which can be a consequence or reflection of systemic and metabolic diseases such as hypertension, diabetes, renal failure, sleep apnea and obesity in addition to local processes such as atrial wall stretch, myocardial infarction and genetic factors.^7 , 8^ The inflammatory reaction that involves oxidative stress, alterations in calcium regulation, production of pro-inflammatory cytokines, proliferation of fibroblasts and myofibroblasts as well as extracellular matrix and apoptosis causes atrial fibrosis, revealed on the electrocardiogram by the prolongation of the PR or AVB interval and increased in the atrial diameter and volume on echocardiography.^9^

In order to assess the prognostic factor of all AVBs in a Latino population, Paixão et al. from the CODE (Clinical Outcomes in Digital Electrocardiology) study evaluated the association between AVB and overall mortality in a Brazilian cohort of primary care, with 1,557,901 patients, with a mean follow-up of 3.7 years, based on a database with electrocardiograms performed mostly in primary health units. Of these, 40% were men, and the mean age was 51. The prevalence of AVB was 1.38%, the majority of the first degree (1.32% - 20,644), with 0.02% (273) and 0.04% (621) of the second and third degree, respectively. Patients with first, second and third degree AVB was associated with 24% (RS= 0.76; 95% CI: 0.71 to 0.81; p < 0.001), 55% (RS = 0.45; 95% CI: 0.27 to 0.77; p = 0.01) and 64% (RS = 0.36; 95% CI: 0.26 to 0.49; p < 0.001) lower survival rate when compared to the control group, respectively, and only Mobitz I AVB (212 patients), in the analysis of survival divided by AVB subtype, were not associated with higher mortality, unlike patients with AVB 2:1 (61 patients), with a 79% lower survival rate than the control group. Beside worst prognosis, with the lowest survival, in patients with second-degree (except Mobitz I) and third-degree AVB, the study reaffirmed the reduction in survival in patients with first-degree AVB.^10^ It is worth mentioning that the mean age was similar to other studies (56 years old) that showed similar outcomes concerning first-degree AVB in a systematic review and meta-analysis carried out by Kwok et al.^1^ with 400,750 patients in which they observed an increase in the relative risk of 1, 24 (95% CI 1.02-1.51) for mortality, 1.39 (95% CI 1.18-1.65) for heart failure and 1.45 (95% CI 1.23-1.71) for AF. Interestingly, there was no increase in cardiovascular mortality in this meta-analysis, data not evaluated by the CODE study. Another particularity in the Brazilian cohort is the relatively frequent presence of Chagas disease, a frequent cause of AVB.

With current evidence, the first-degree AVB should be viewed more carefully, and the electrocardiogram, despite all the advances in cardiology, with increasingly detailed and specific diagnostic imaging tests, remains a simple, available, useful, and fundamental tool in our routine.
